# Genome sequencing and phylogenetic analysis of allotetraploid *Salix matsudana* Koidz

**DOI:** 10.1038/s41438-020-00424-8

**Published:** 2020-12-01

**Authors:** Jian Zhang, Huwei Yuan, Yujuan Li, Yanhong Chen, Guoyuan Liu, Meixia Ye, Chunmei Yu, Bolin Lian, Fei Zhong, Yuna Jiang, Jichen Xu

**Affiliations:** 1 Key Lab of Landscape Plant Genetics and Breeding, School of Life Science, Nantong University, 226019 Nantong, China; 2 State Key Laboratory of Subtropical Silviculture, Zhejiang A&F University, 311300 Hangzhou, China; 3 Jiangsu Riverine Institute of Agricultural Sciences, 226541 Nantong, Jiangsu China; 4 College of Biological Sciences and Technology, Beijing Forestry University, 100083 Beijing, China; 5 National Engineering Laboratory of Tree Breeding, Beijing Forestry University, 100083 Beijing, China

**Keywords:** Comparative genomics, Plant breeding

## Abstract

Polyploidy is a common phenomenon among willow species. In this study, genome sequencing was conducted for *Salix matsudana* Koidz (also named Chinese willow), an important greening and arbor tree species, and the genome of this species was compared with those of four other tree species in Salicaceae. The total genome sequence of *S. matsudana* was 655.72 Mb in size, with repeated sequences accounting for 45.97% of the total length. In total, 531.43 Mb of the genome sequence could be mapped onto 38 chromosomes using the published genetic map as a reference. The genome of *S. matsudana* could be divided into two groups, the A and B genomes, through homology analysis with the genome of *Populus trichocarpa*, and the A and B genomes contained 23,985 and 25,107 genes, respectively. 4DTv combined transposon analysis predicted that allotetraploidy in *S. matsudana* appeared ~4 million years ago. The results from this study will help reveal the evolutionary history of *S. matsudana* and lay a genetic basis for its breeding.

## Introduction

Willows, the generic term for tree species in *Salix*. L and *Chosenia* Nakai of Salicaceae, include deciduous arbors and shrubs mainly distributed in temperate and frigid zones of the Northern Hemisphere. Willow tree species play important roles in energy production, afforestation, and greening. To date, over 520 willow tree species have been discovered worldwide. Asia is the center of origin for willow, and 257 willow tree species are distributed in China^1,2^. Willow tree species are resistant to a number of stresses, including salt stress^3^, drought stress^4^, water stress^5^, and heavy metal stress^6^, and provide important functions in ecological restoration^7^.

The genetic background of willow is complex. Chromosome ploidy, including diploidy (2*n* = 12x = 38), triploidy, tetraploidy, pentaploidy, hexaploidy, and even dodecaploidy (2*n* = 12x = 228), is abundant among willow species in nature^8,9^. Most shrub willow tree species are diploid, while most arbor species are allopolyploid^10–12^. To provide the necessary genetic characteristics of willow for scientific study, the genomes of several diploid shrub willow species, including *Salix suchowensis*^13^ and *Salix purpurea* (available at http://phytozome.jgi.doe.gov), were sequenced successively.

*Salix matsudana* Koidz is an allotetraploid arbor tree species in *Salix* with high stress resistance and strong adaptability. *S. matsudana* has been cultivated widely in China, and has thus been given the name “Chinese willow”. *S. matsudana* has been introduced to many areas around the world, including Australia, Europe, and North America^14^. Selection and breeding of new stress-resistant varieties of *S. matsudana* have been conducted at several institutions, and a series of elite varieties, including “9901” and “Hailiu 1”, were selected and applied in coastal regions of China. To better understand the molecular characteristics of *S. matsudana*, the first draft genome of *S. matsudana* was assembled, and the phylogenetic characteristics of *S. matsudana* were revealed by comparing its genome with available genomes of *S. suchowensis*, *S. purpurea*, *Populus trichocarpa*^15^, and *Populus euphratica*^16^ in the same family.

## Results

### Genome assembly and quality assessment

According to the kmer distribution, the heterozygosity rate of *S. matsudana* was estimated to be 0.71% (Supplementary Fig. S1), demonstrating the highly heterozygous genome of this species. In total, 78.50 G of raw data with a depth of 125× and Q30 of >93.11% was obtained by the second-generation Illumina sequencing platform (Supplementary Table S1); 37.58 G of raw data with a total base content of 35.64 Gb, a mean length of 10.31 Kb, and a mean read quality of 0.834 were obtained by the third-generation PacBio sequencing platform (Supplementary Table S2), and 289 G of optical data were obtained based on standard protocols. The raw data from the third-generation PacBio sequencing platform were initially assembled using Canu^17^ and Quickmerge^18^ software, followed by error correction with Pilon^19^ software using the second-generation sequencing data, and final scaffold extension with Irys-scaffolding^20^ software using the optical data. Finally, a genome of *S. matsudana* with a total size of 655.72 Mb was obtained, for which the number of scaffolds, scaffold N50, contig N50, and GC content were 2120, ~12.35 Mb, ~945.75 Kb, and 33.55%, respectively (Table 1).

**Table 1:** Summary of the *S. matsudana* genome

Scaffold number	Scaffold length (bp)	Scaffold N50 (bp)	Scaffold N90 (bp)	Scaffold max (bp)	Gap total length (bp)
2120	655,719,782	12,349,754	180,627	28,420,164	726
Contig number	Contig length (bp)	Contig N50 (bp)	Contig N90 (bp)	Contig max (bp)	GC content (%)
2819	653,522,500	945,757	148,606	5,826,838	33.55

Note: contig number: the number of contigs >1 Kb in length; contig length (bp): the length of contigs >1 Kb in length; contig N50 (bp): the length of contigs with an N50 > 1 Kb; contig N90 (bp): the length of contigs with an N90 > 1 Kb; contig max (bp): the length of the longest contig >1 Kb in length; gap total length (bp): total length of the gap

After quality control of the assembled sequences, 450 core genes accounting for 98.25 of the total (458; Supplementary Table S3) and 1313 complete core genes accounting for 94.38 of the total (1440; Supplementary Table S4) were identified in the CEMGA (Core Eukaryotic Genes Mapping Approach) database and BUSCO database, respectively, demonstrating the acceptable consecutiveness, coverage rate, and accuracy of the *S. matsudana* genome constructed in this study.

### Genome annotation

#### Analysis of repeated sequences

A repeated sequence database of the *S. matsudana* genome was constructed based on the principles of structure prediction and de novo sequencing. The genome of *S. matsudana* was clustered and ranked according to a previously published genetic map^21^. In total, 531.43 Mb of the genome sequences (accounting for 81.43%) was mapped onto the 38 chromosomes of *S. matsudana*, the length of which ranged from 8.80–28.42 Mb. In total, 300.41 Mb of repeated sequences, accounting for 45.97% of the whole genome, was identified, among which the numbers of SSRs and unknown repeated sequences were 6185 and 116,059, accounting for 0.51% and 6.76% of the total genome, respectively (Supplementary Table S5).

### Gene prediction

**Fig. 1:**
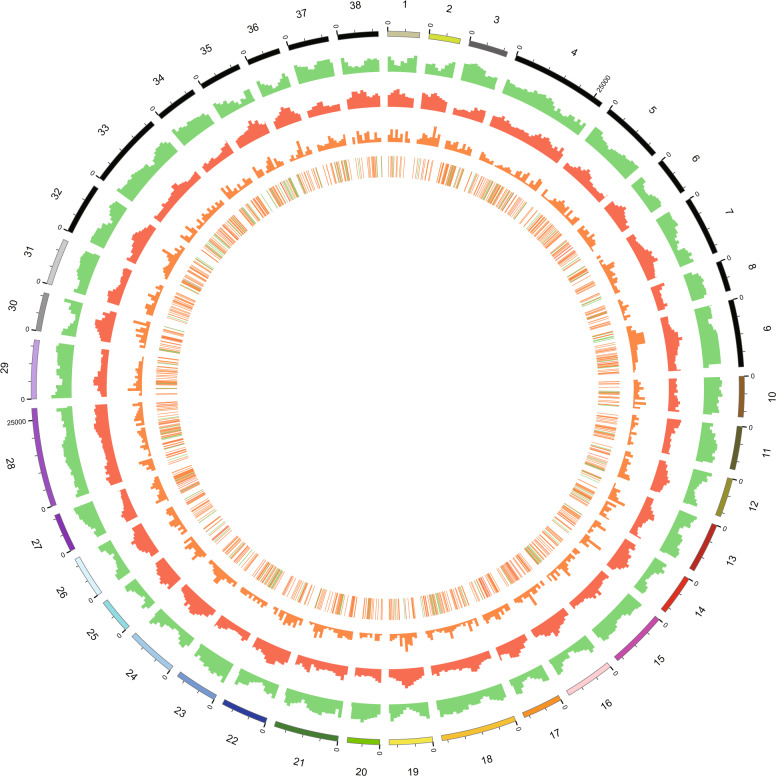
Circular graph of the *S. matsudana* genome. The outer ring represents the size of individual chromosomes, with a scale of 1 Mb; the second ring (green) represents the distribution of genes; the third ring (red) represents the distribution of repeated sequences; the fourth ring (orange) represents the distribution of pseudogenes; and the fifth ring represents the distribution of tRNAs (orange), miRNAs (green), and rRNAs (red)

A total of 57,841 genes with a total length of 216,437 Kb, a mean exon length of 229 bp, a mean intron length of 350 bp, and a density of 108.9 genes per Mb were predicted. In addition, 220 miRNAs (belonging to 27 gene families), 321 rRNAs (belonging to 4 gene families), 1557 tRNAs (belonging to 23 gene families), and 3494 pseudogenes were discovered (Fig. 1). The results from blast alignment showed that 99.49% of the genes were annotated in the NR, COG, and KEGG databases (Supplementary Table S6), while only 295 genes were not annotated in these databases.

### Comparisons between genomes of willow and poplar

Comparisons of genome sequences between *S. matsudana* and four other species in Salicaceae, namely, *P. trichocarpa*, *P. euphratica*, *S. suchowensis*, and *S. purpurea*, showed that the total number of genes in *S. matsudana* were 1.53 times and 2.17 times that in *S. purpurea* and *S. suchowensis*, respectively (Table 2).

**Table 2:** Comparisons between the genomes of five tree species in Salicaceae

	*S. purpure*a (diploid)	*S. suchowensis* (diploid)	*S. matsudana* (tetraploid)	*P. euphratica* (diploid)	*P. trichocarpa* (diploid)
Genome size	392 Mb	425 Mb	656 Mb	497 Mb	485 Mb
Assembled genome sequence size	348 Mb	303 Mb	531 Mb	323 Mb	388 Mb
Scaffold N50	190.9 Kb	925.0 Kb	12,349.8 Kb	482.1 Kb	19,500 Kb
Contig N50	45.6 Kb	17.4 Kb	945.8 Kb	40.4 Kb	552.8 Kb
Number of predicted genes	37,865	26,599	57,841	34,279	42,950
Gene density (genes per Mb)	109	88	109	106	111

**Fig. 2:**
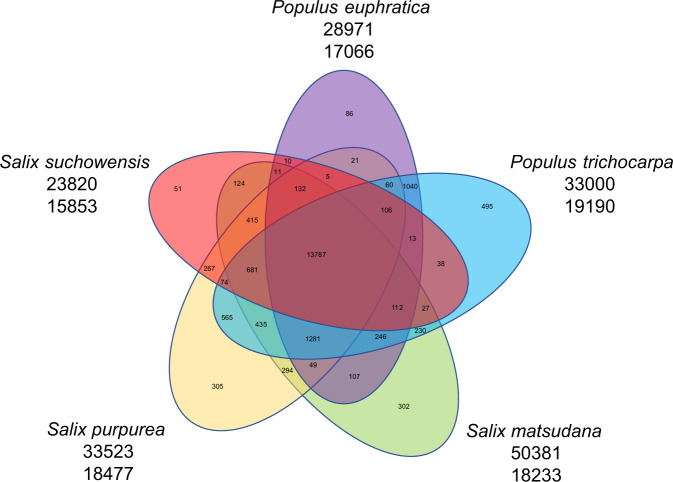
The common and specific gene families in the genomes of five tree species in *Salicaceae*. The numbers under the five tree species represent cluster gene number and total gene family number, respecitively

A total of 50,381 of the 57,841 genes in the *S. matsudana* genome could be clustered into 18,233 gene families, among which 13,787 were common among the five species of Salicaceae, while 302 were specific to *S. matsudana* (Supplementary Table S7 and Fig. 2). A total of 793 genes were included in the gene families specific to *S. matsudana*, and their functions were related to metabolic process, cellular process, response to stimulus, and biological regulation (Supplementary Fig. S2).

### Phylogenetic analysis of *S. matsudana*

#### Expansion analysis of genes in *S. matsudana*

Further investigation of the expansion and contraction of gene families in the five tree species in Salicaceae demonstrated that the numbers of gene families and genes that were expanded in *S. matsudana* were 3128 and 20,699, respectively (Supplementary Fig. S3). The functions of these genes were mainly related to metabolic process, cellular process, cell part, cell binding, catalytic activity, and so on. In addition, a series of root development-related gene families, such as root cap, root hair defective 3 GTP-binding protein (*RHD3*), and transcription factor regulating root and shoot growth via Pin3, were identified as expanded families, and their gene numbers were expanded to 18, 27 and 29, respectively.

#### Fourfold degenerate site (4DTv) analysis of the five tree species in Salicaceae

**Fig. 3:**
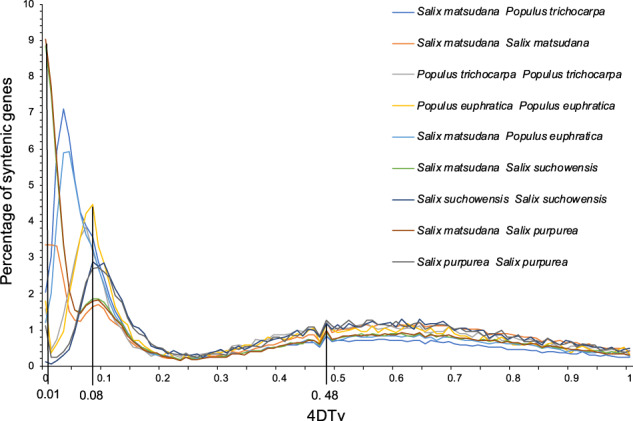
The 4DTv distribution among the five tree species in *Salicaceae*. The x-coordinates and y-coordinates represent 4DTv distance and percentage of syntenic genes, respectively

The duplication time of the genome was estimated by comparing the 4DTv mutation rates among genes in the collinear fragments of the five tree species in Salicaceae (Fig. 3). A genome-wide replication event was predicted to have occurred 220–358 million years ago (MYA) among the ancestors of Salicaceae, since the first peak was at 0.48 and the synonymous mutation rates (*R* values) of Salicaceae ranged from 1.09 × 10^−9^ to 0.67 × 10^−9^/site/year^13^. Similarly, a second genome-wide replication event was predicted to have occurred 36.7–59.7 MYA among species in Salicaceae, since the second peak was at 0.08, at which time differentiation between poplar and willow was estimated to have appeared. In addition, the allotetraploid was estimated to have formed 4.6–7.5 MYA in *S. matsudana*, since a peak appeared at 0.01 for this species.

#### Long terminal repeat sequence analysis among the five tree species in Salicaceae

Among the long terminal repeat sequence (LTR) transposons in *S. matsudana*, 213,911, with a total length of 93,513,152 bp and accounting for 14.31% of the repeated sequences, were Copia transposons, while 227,569, with a total length of 100,397,354 bp and accounting for 15.36% of the repeated sequences, were Gypsy transposons.

**Fig. 4:**
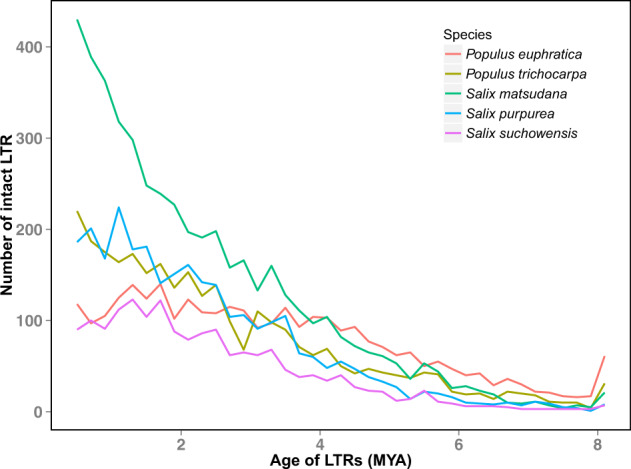
Insertion time estimation for LTR transposons in the five tree species in Salicaceae. The x-coordinates and y-coordinates represent the age of LTRs and number of intact LTR, respectively

After estimation of the two types of LTRs by sequence bifurcation analysis, it was found that the insertion of LTR transposons into Salicaceae genomes began 6 MYA, while the insertion of LTR transposons began 4 MYA in the *S. matsudana* genome (Fig. 4), further confirming that the formation of allotetraploid *S. matsudana* occurred recently.

#### Expression analysis of root-related genes in arbor and shrub willows

**Fig. 5:**
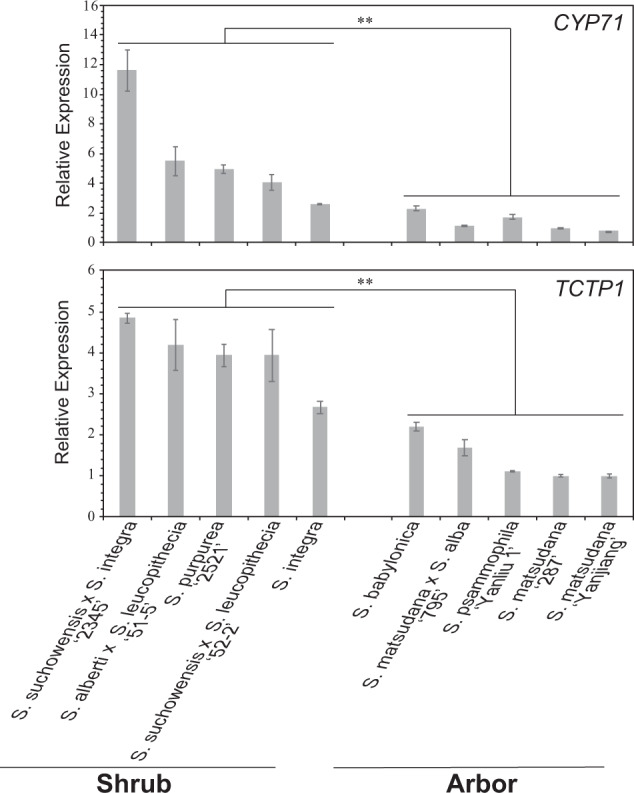
The expression levels of TCTP1 and CYP71 in the roots of different arbor and shrub willow varieties. The x-coordinates and y-coordinates represent the willow varieties and relative expression, respectively

Arbor and shrub willows are diverse in terms of tiller number, plant height, and primary root development. For instance, the primary roots are well developed in arbor willow, while the primary roots are not obvious, but the lateral roots are well developed in shrub willow. A series of genes were identified as rapidly evolving genes and annotated as root-related genes after comparing the two shrub willow genomes with that of *S. matsudana*. The expression levels of these genes were determined in ten arbor and shrub willow varieties, using *SmACTIN1* as a reference gene. The results showed that *TCTP1* and *CYP71* were differentially expressed in the roots of arbor and shrub willows (Fig. 5). Results from *Arabidopsis* showed that *TCTP* was expressed throughout the plant, but with the highest levels in meristematic regions of the shoot and root, demonstrating its functions in lateral root development, root development, and root hair cell tip growth. CYP71 is a WD40 domain cyclophilin that functions in gene repression, organogenesis, and meristem development. Based on the above results, *TCTP1* and *CYP71* were predicted to be related to the differentiation of arbor and shrub willows.

### Analysis of chromosome differentiation and genome comparison in *S. matsudana*

#### Chromosome differentiation in the *S. matsudana* genome

The chromosomes of *S. matsudana* are displayed in a boxplot according to identity value. Among the 38 chromosomes of *S. matsudana*, the chromosome group homologous to that of *P. trichocarpa* was identified and named the A genome, while the remaining 19 chromosomes were classified as the B genome (Supplementary Fig. S4). The A genome was 252.68 Mb in length, with a density of 95 genes per Mb, while the B genome was 278.76 Mb in length, with a density of 90 genes per Mb. The densities of both the A and B genomes of *S. matsudana* were lower than those in *P. trichocarpa*.

**Fig. 6:**
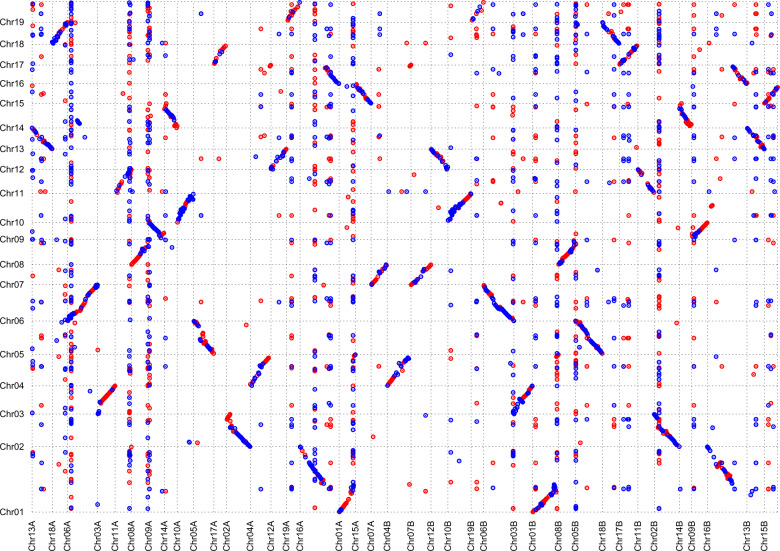
The collinearity between the genomes of *S. matsudana* and *P. trichocarpa*. The *x*-coordinates and *y*-coordinates represent the genomes of *S. matsudana* and *P. trichocarpa*, respectively. Red represents positive alignment, while blue represents reverse alignment

The collinearity between the genomes of *S. matsudana* and *P. trichocarpa* was analyzed by MUMMER software. The results showed that the recombination events occurred between Chr1 in *P. trichocarpa*, and Chr1A and Chr16A in the A genome of *S. matsudana*, and similar events were also observed for the B genome of *S. matsudana* (Fig. 6), demonstrating that Chr1 in *P. trichocarpa* underwent a breakage event. One of the fragments was reconnected with Chr16 and formed Chr1 in *S. matsudana*, and the other fragment formed Chr1 in *S. matsudana*, which was consistent with the findings of previous studies^22,23^.

#### Comparisons between different genome groups of *S. matsudana*

**Fig. 7:**
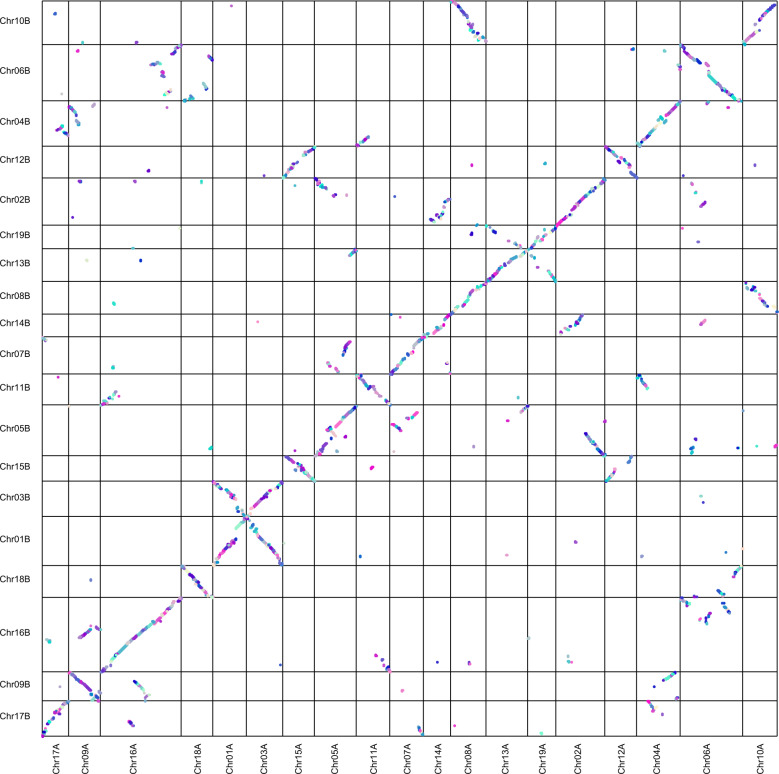
The collinearity relationships between the A and B genomes of *S. matsudana*. The *x*-coordinates and *y*-coordinates represent the A and B genomes of *S. matsudana*, respectively

Comparisons between different genome groups of *S. matsudana* showed that the number of genes in the A and B genomes were 23,985 and 25,107, respectively. Collinearity analysis between the A and B genomes revealed different degrees of rearrangement between chromosomes in the A and B genomes, such as between Chr1A and Chr3B, and between Chr1B and Chr3A. In addition, duplications of some chromosome fragments were also observed. For example, Chr8A in the A genome, corresponding to Chr8B in the B genome, was duplicated into Chr10B (Fig. 7).

PAVs between the A and B genomes of *S. matsudana* were initially identified by ppsPCP^24^ software, further verified by searching for PAV sequences in the reference genome, and finally confirmed by comparing the PAVs with queries. The results indicated that the number of PAV mutation-related genes in the A genome compared with the B genome was 19,841, while the number of PAV mutation-related genes in the B genome compared with the A genome was 12,246. GO enrichment analysis of the genes with PAVs between the A and B genomes suggested that the functions of these genes were consistent in rank between the A and B genomes in terms of biological process, cellular component, and molecular function (Supplementary Table S8 and Supplementary Fig. S5). For example, most of the genes with PAVs, i.e., 3693 in the A genome and 4231 in the B genome, were annotated as being associated with the metabolic process function in the biological process category, accounting for 54.28% and 55.66% of the total genes in the corresponding A and B genomes, respectively.

KEGG enrichment analysis of the genes with PAVs between the A and B genomes suggested that nine of the top ten significantly enriched pathways were different between the A and B genomes. The most enriched pathways were the pyruvate metabolic pathway, and the starch and sucrose metabolic pathway in the A and B genomes, with 40 and 100 genes annotated, respectively (Supplementary Fig. S6). Significant differences between the A and B genomes suggested their varying roles in regulating basal metabolism.

#### Genome differentiation of willow

A phylogenetic tree was constructed according to the single-copy protein sequences in the five tree species in Salicaceae and the differentiation time of Salicaceae^13^. The differentiation time of the two shrub willows was ~13.3 MYA, while the differentiation of the original species conferring the A and B genomes to *S. matsudana* started ~25.2 MYA (Supplementary Fig. S7).

## Discussion

### Ploidy analysis of willow chromosomes

Willows, the generic term for landscaping and energy tree species in Salicaceae, are variable in terms of chromosome ploidy, from 2*n* = 38 to 12*n* = 228 (refs. ^8,9^). Most shrub willows are diploid, with a genome size of ~400 Mb. For instance, the genome sizes of *Salix triandra* L. (ref. ^25^) and *S. suchowensis* Cheng^13,26^. are 386 and 425 Mb, respectively. Arbor willows are mainly allopolyploid^10–12^, with a genome size of >650 Mb. For example, the genome sizes of *Salix babylonica* L. (ref. ^27^) and *Salix alba* L. (ref. ^25^) are 748 and 807 Mb, respectively. *S. matsudana* is a tetraploid species^28^. A previous study by our group estimated that the genome size of *S. matsudana* was ~670 Mb by flow cytometry^29^. In this study, the genome size of this species was 656 Mb (Table 1), which was in accordance with the size in the previous study.

Chromosome doubling of somatic cells and unreduced germ cells are the main pathways for the formation of polyploids in plants during evolution^30^. Autopolyploids are polyploids derived from the same species, while allopolyploids are polyploids originating from different species^31^. For example, hexaploid bread wheat (*Triticum aestivum* L., AABBDD) originated initially from a hybridization event between *Triticum urartu* (AA) and *T. aestivum* L. (BB) to form the tetraploid *Triticum turgidum* (AABB), followed by the hybridization event between *Aegilops tauschii* (DD) and *T. turgidum*^32^. The origin of tetraploid *Gossypium hirsutum* L. (AADD) and *Gossypium barbadense* L. (AADD) was dated back to 1.5 MYA, which involved the formation of the A genome donated by *Gossypium herbaceum* L. or *Gossypium arboreum* L., and the hybridization event between the A genome and D genome (donated by *Gossypium raimondii* Ulbrich)^33^. The genome comparison in the present study suggested that 19 of the 38 total chromosomes in *S. matsudana* were consistent with those in the *P. trichocarpa* genome (Fig. 6), indicating that *S. matsudana* might have originated from hybridization between the ancestors of *P. trichocarpa* and another species. 4DTv combined transposon analysis predicted that the genome of *S. matsudana* doubled ~4 MYA (Figs. 3 and 4), indicating that the formation of the allotetraploid in this species might have occurred during this period.

### Split between arbor and shrub willows

Arbors and shrubs are two growth forms specific to tree species. Different types of arbors and shrubs have been observed simultaneously in some willow species^34^. In this study, the split between the two shrub willow species *S. suchowensis* and *S. purpure*a was estimated to have occurred ~13.3 MYA, while the split between the species donating the A genome in *S. matsudana* and shrub willow occurred ~19.0 MYA (Supplementary Fig. S6). However, because of the limited number of willow genomes sequenced, the exact split time between arbor and shrub willows needs to be further studied.

Arbor and shrub willows displayed significant phenotypic variations in terms of root, branch, and crown growth. The differences in root growth between arbor and shrub willows have been rarely studied, using molecular biology methods. In the current study, *TCTP1* and *CYP71*, screened from rapidly evolving genes of the two shrub willows in comparison to *S. matsudana*, were identified as differentially expressed genes between the roots of arbor and shrub willow varieties. It was reported that *TCTP1* functioned in lateral root development and determined total root length in *Arabidopsis*^35^. In addition, *TCTP* induces the formation of adventitious roots in tobacco^36^. *CYP71* functions in gene repression and organogenesis, and abnormalities in *CYP71* result in the ectopic activation of homologous genes regulating meristem development. Mutants of *CYP71* displayed phenotypes of low apical meristem activity and disrupted root growth^37^. *TCTP1* and *CYP71* were highly expressed in the shrub willow varieties, but weakly expressed in the arbor willow varieties (Fig. 5), suggesting that they might have been involved in the split between arbor and shrub willows. Further functional analysis of these genes would provide insight into the nature of the split between arbor and shrub willows.

### Differentiation time between the A and B genomes of *S. matsudana*

Most species have undergone >2 whole-genome duplications (WGDs), which facilitated their survival and adaptation to different environmental conditions^38^. The results from this study indicated that species in Salicaceae had undergone at least two WGD events, and the second WGD occurred 36.7–59.7 MYA (Fig. 3).

Fossil records and genome sequences are commonly used to estimate the time of species differentiation. Poplar and willow belong to the same family but different genera. Previous studies based on fossil records revealed that the differentiation of Salicaceae occurred ~45–48 MYA^39–41^. The differentiation time of Salicaceae was estimated to be 12 (ref. ^42^), 48 (ref. ^41^), and 52 MYA^13^ based on sequencing results for the chloroplast, plastid, and nuclear genomes of *S. suchowensis*, respectively. The variation in differentiation time might be caused by differences in sample selection, which could lead to variation in the correction coefficient and even the final result. The differentiation of the A and B genomes of *S. matsudana* (Supplementary Fig. S7) was deduced to be 25.2 MYA based on the split time of Salicaceae estimated by the *S. suchowensis* genome, as well as the single-copy orthologs within the whole genome.

## Methods

### Genome sequencing

Fresh leaves of *S. matsudana* were collected in Changjiang town, Rugao city, Jiangsu Province, China, frozen in liquid nitrogen immediately, and stored at −80 °C for subsequent genomic DNA extraction and library construction. The genomic DNA was fragmented by g-TUBE, repaired, ligated with a dumbbell-shaped adaptor, and digested with exonuclease. The target fragments were screened by BluePippin and used for library construction. Second-generation sequencing was performed according to the protocols provided by the Illumina company. Assembly of the genome sequences from the third-generation sequencing was conducted by the PacBio technique. All sequencing procedures were performed by the BioMarker Technologies Company (Beijing, China).

### Genome annotation

#### Annotation of repeated sequences

The repeated sequence database of the *S. matsudana* genome was constructed initially based on the structure prediction and de novo prediction, using LTR FINDER^43^, MITE-Hunter^44^, RepeatScout^45^, and PILER-DF^46^ software. The database was classified with PASTEClassifier^47^ and combined with the Repbase^48^ database to form the final repeated sequence database. The repeated sequences were annotated based on the final repeated sequence database using RepeatMasker^49^ software.

#### Gene expression

Three strategies, namely, de novo prediction, homologous species-based prediction, and unigene-based prediction, were adopted for gene prediction. The prediction results from different strategies were integrated by EVM^50^ software. GENSCAN^51^, Augustus^52^, GlimmerHMM^53^, GeneID^54^, and SNAP^55^ were used for de novo prediction. *P. trichocarpa* and *P. euphratica* were selected for homologous species-based prediction using GeMoMa^56^. PASA^57^ was used for unigene-based prediction.

#### Annotation of gene functions

The predicted gene sequences were subjected to BLAST^58^ alignment with the NR^59^, COG^60^, and KEGG^61^ functional databases. Functional annotations in the GO^62^ database were compared with the NR database alignment result using Blast2GO^63^. In addition, the predicted genes were also subjected to COG, KEGG, and GO enrichment analyses.

### Comparative genomic analysis

The protein sequences of *S. matsudana* were aligned with those in related species, including *S. suchowensis*, *S. purpurea*, *P. trichocarpa*, and *P. euphratica*, to analyze gene duplication within species, gene evolution between species and the classification of species-specific genes. Clustering analysis of gene families was conducted by OrthoMCL^64^ software. The 4DTv mutation rate was calculated for each of the homologous gene pairs between and within species. LTR sequences with scores ≥6 were detected in the genome by LTR FINDER^43^ and PS SCAN^65^, but the repeated sequences detected by LTR FINDER were filtered. The flanking sequences of LTRs were extracted and aligned with MUSCLE^66^. The degrees of sequence differences were calculated by DistMat software, with the Kimura model and a 7.3 × 10^−9^ molecular clock.

### qRT-PCR of *CYP71* and *TCTP1*

The branches of “2345” (*S. suchowensis* × *S. integra*), “51-5” (*S. alberti* × *S. leucopithecia*), “2521” (*S. purpurea*), “52-2” (*S. suchowensis* × *S. leucopithecia*), *S. integra, S. babylonica*, “Yanliu 1” (*S. psammophila*), “795” (*S. matsudana* × *S. alba*), “287” and “Yanjiang” (*S. matsudan*a) were collected and clipped to a length of 10 cm and diameter of 1 cm. Three biological replicates with ten cuttings per replicate were analyzed for each variety. After 15 days of hydroponic culture, the roots of each cutting were collected and stored at −80 °C. Total RNA from the roots of the ten varieties was extracted using an RNAprep Pure Plant kit (Tiangen). The expression levels of the two genes (*CYP71* and *TCTP1*) in each biological replicate were measured by qRT-PCR using three technological replicates. A PrimeScript^TM^ RT Reagent Kit (Takara) was used for reverse transcription of the RNAs. Reverse transcription was performed at 37 °C for 30 min and 85 °C for 5 s. A One-Step SYBR Primer Script PLUS RT-PCR Kit (Takara) was used for qRT-PCR of the *CYP71* and *TCTP1* genes. The reaction was performed at 95 °C for 2 min, followed by 40 cycles of 95 °C for 5 s, 56 °C for 30 s, and 72 °C for 20 s. The *Actin* gene in *Salix* was used as the reference gene. The qRT-PCR primers are listed in Supplementary Table S9. Relative expression was measured using the 2^−ΔΔCt^ method. The expression level of “287” (*S. matsudana*) was selected as the reference level (with a relative expression of 1.0).

### Construction of the phylogenetic tree

The A and B genomes of *S. matsudana*, and the genomes of *S. suchowensis*, *S. purpurea*, *P. trichocarpa*, and *P. euphratica* were used for phylogenetic analysis. The sequence of each single-copy gene family was aligned with MAFFT (v7.205), while the regions with significant differences were removed by Gblocks (v0.91b). Finally, supergenes were obtained by connecting all the aligned gene families in each species. ModelFinder in IQ-TREE was used for model testing, and the best model was predicted to be JTT + F + I + G4. The maximum likelihood method was used to construct a rooted phylogenetic tree. *P. trichocarpa* was selected as the outgroup species, and the number of bootstraps was set to 1000. Split times and the gradient and Hessian parameters were calculated by MCMCTREE in PAML (v4.9i) software. Visualization of the phylogenetic tree with split times was performed by MCMCTreeR.

### Collinearity analysis between the A and B genomes of *S. matsudana*

The similar gene pairs, with the criteria of an *E* value < 1e − 5 and a *C* score > 0.5 (*C* scores were filtered by JCVI software), were identified by comparing gene sequences between species using Diamond (v0.9.29.130). gff3 was used to determine whether similar gene pairs were adjacent in the genome.

## Supplementary information

Supplemental materials-revised
