# Supplementary material for: Genome sequencing and phylogenetic analysis of allotetraploid Salix matsudana Koidz
Source: Hortic Res. 2020 Dec 1;7:201. doi: 10.1038/s41438-020-00424-8 (PMC7705746; doi:10.1038/s41438-020-00424-8)
Supplement: Supplementary file 1 — Supplemental materials-revised [file 41438_2020_424_MOESM1_ESM.docx]

**Supplemental Materials**

**
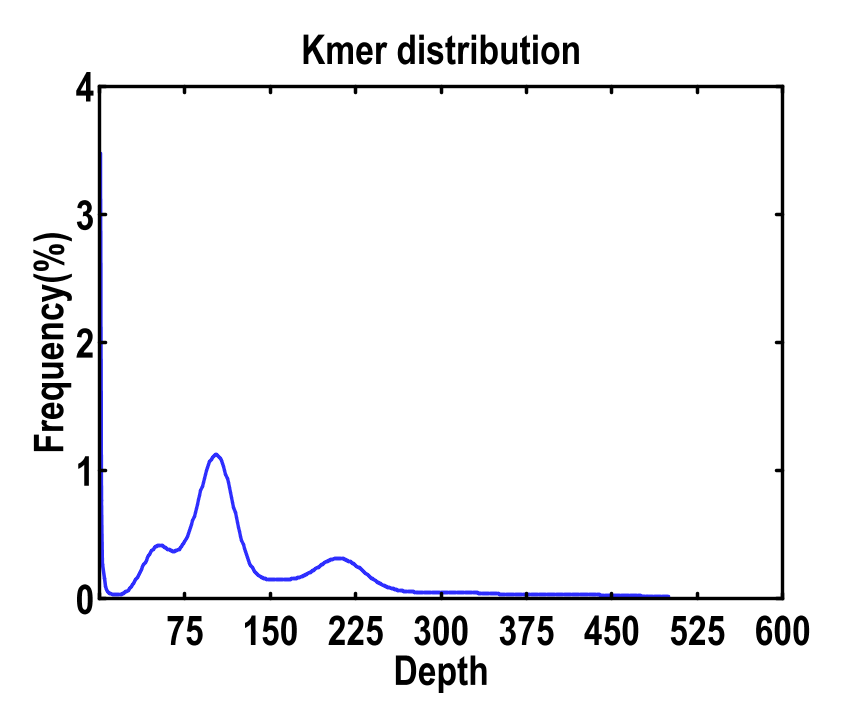
**

**Fig. S1 The Kmer distribution**

**
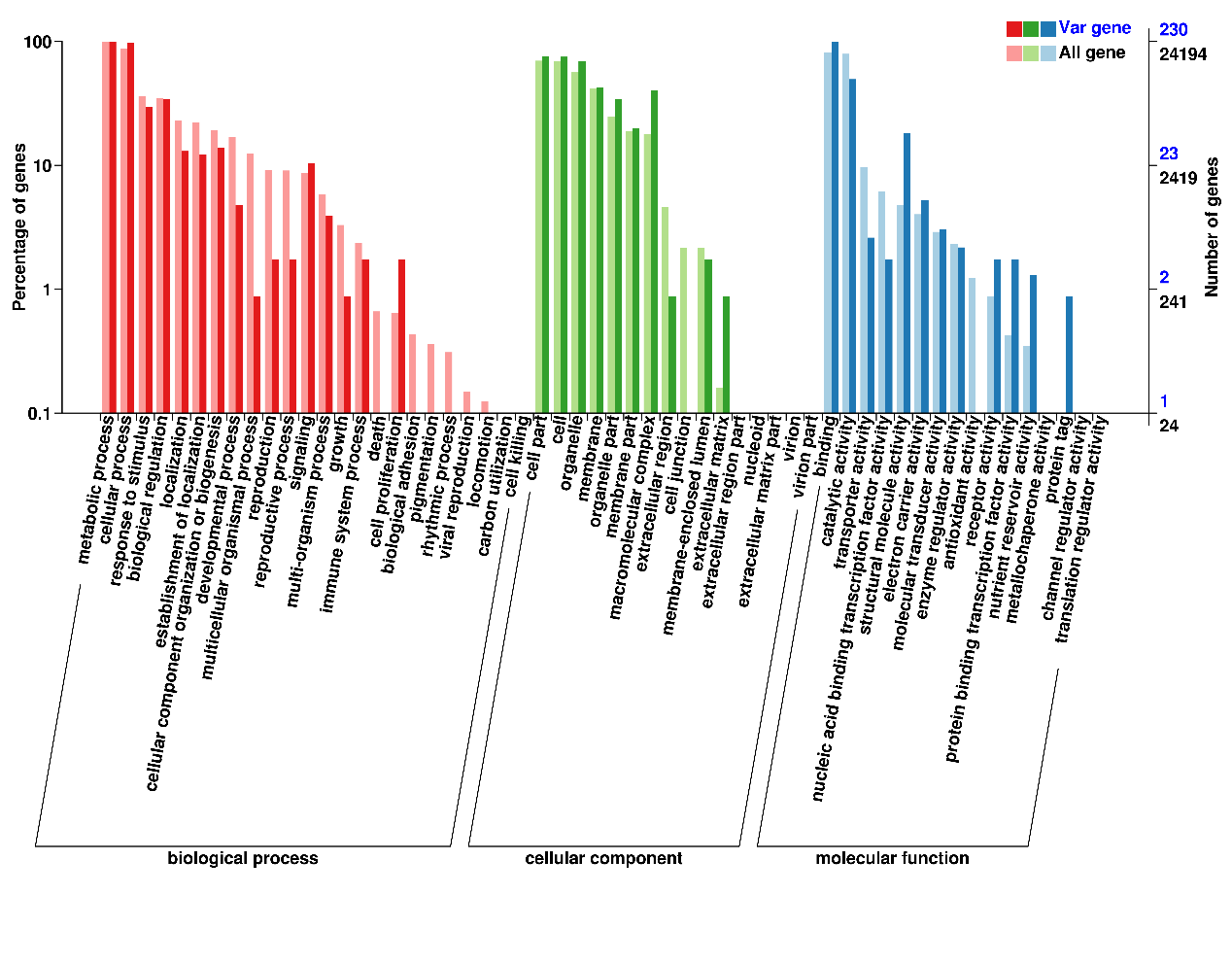
**

**Fig. S2** **GO enrichment analysis of the family genes specific to *S. matsudana***

**
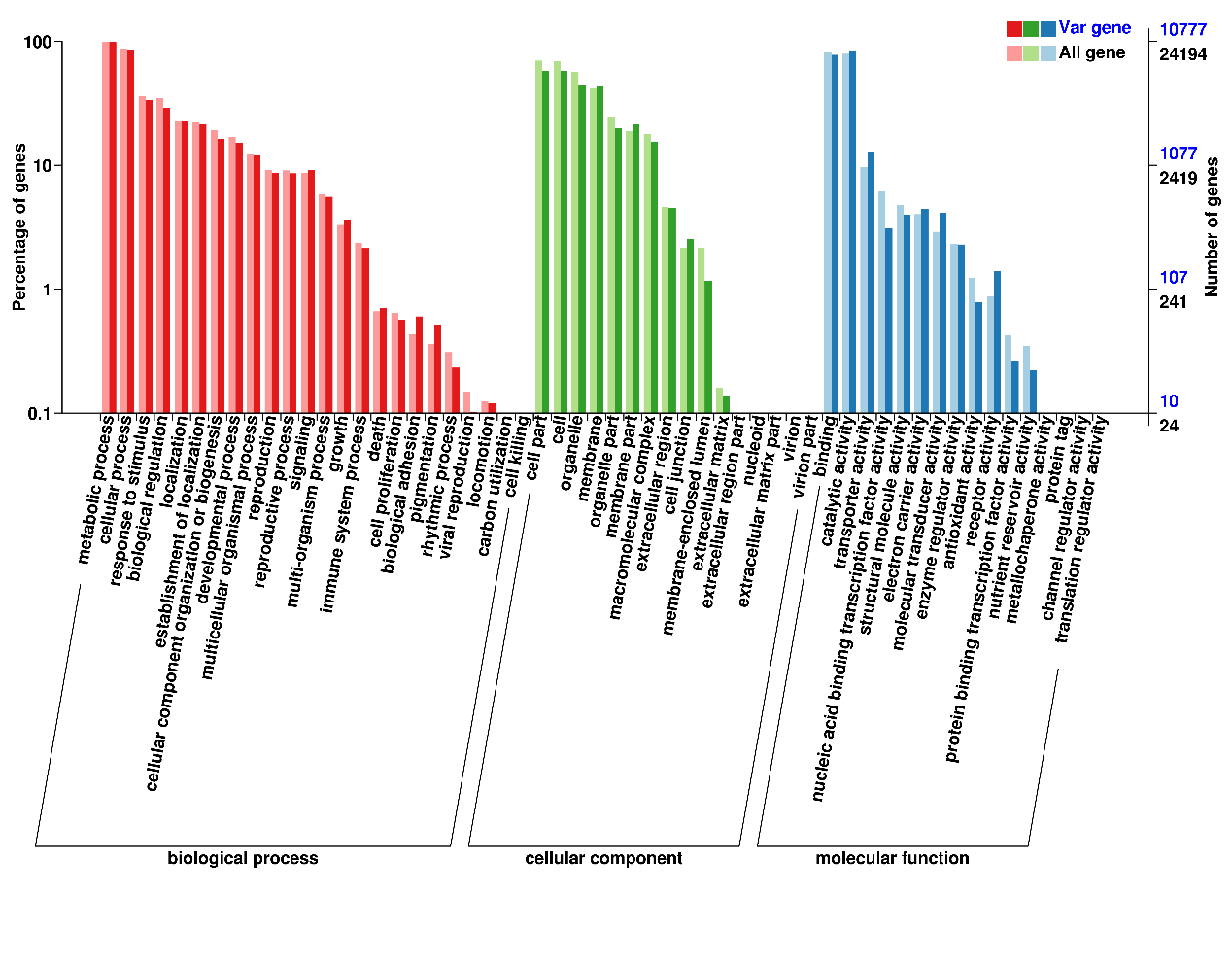
**

**Fig. S3** **GO enrichment analysis of the expansion family genes in *S. matsudana***

**
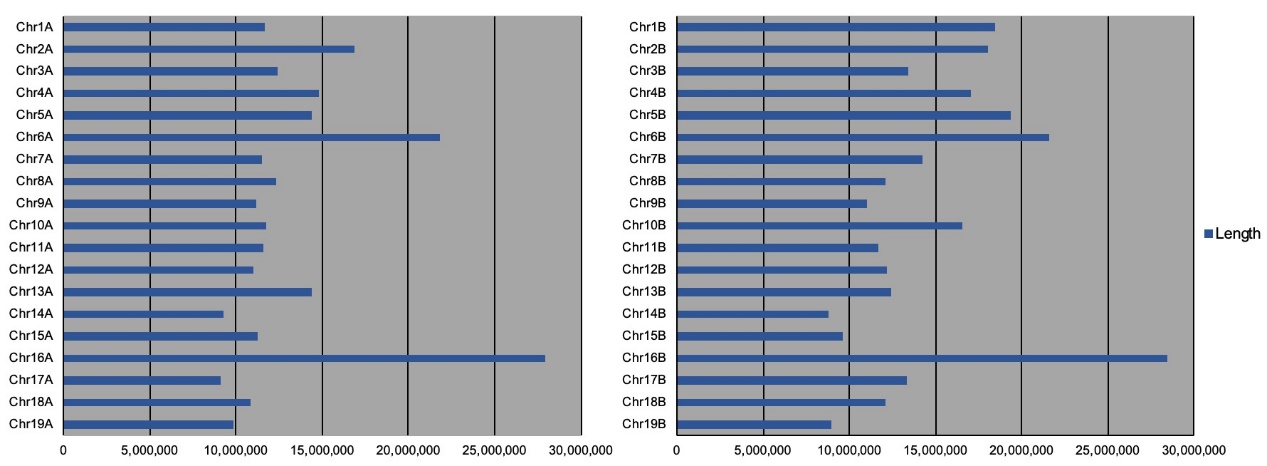
**

**Fig. S4** **The arrangement and length of the A- and B-genome in *S. matsudana***

**
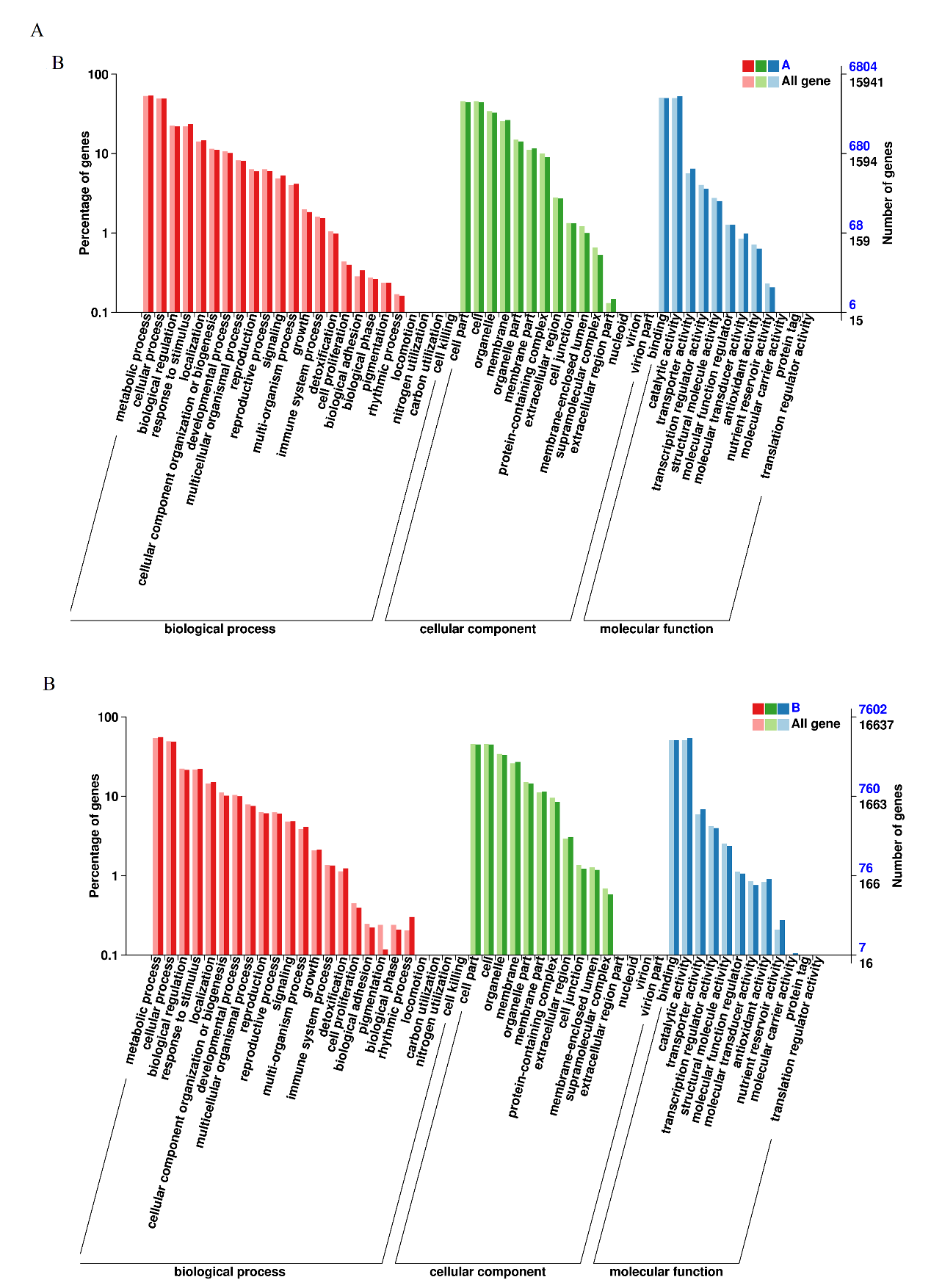
**

**Fig. S5** **GO enrichment analysis of the PAV differential genes in the A- and B-genome of *S. matsudana***

**
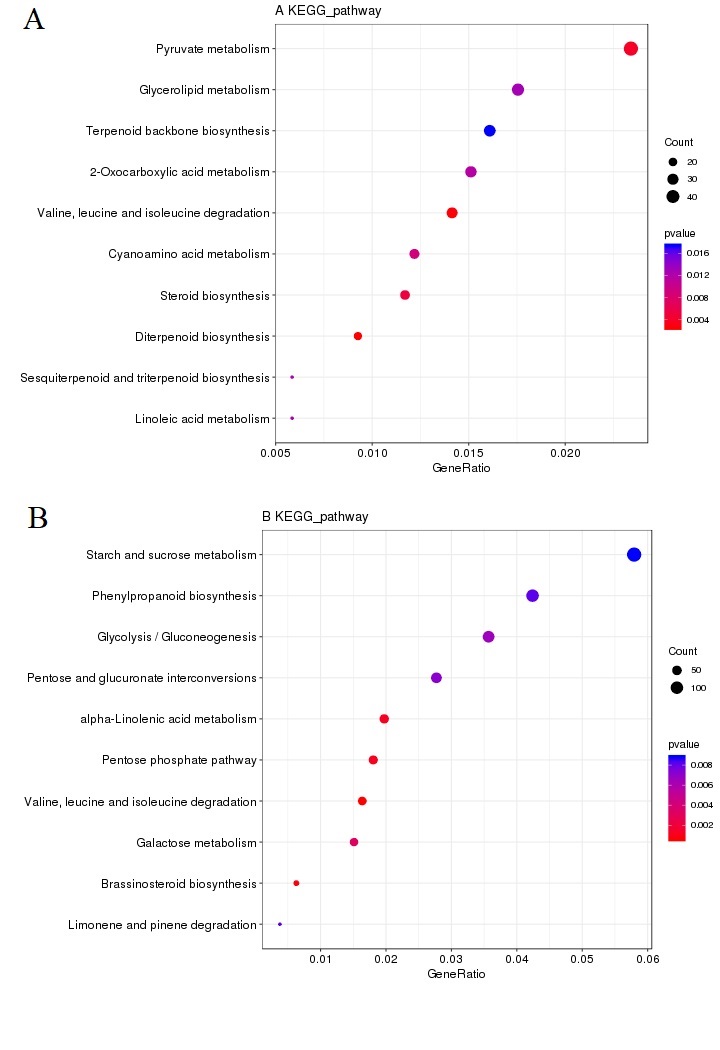
**

**Fig. S6** **KEGG enrichment analysis of the PAV differential genes in the A- and B-genome of *S. matsudana***

**
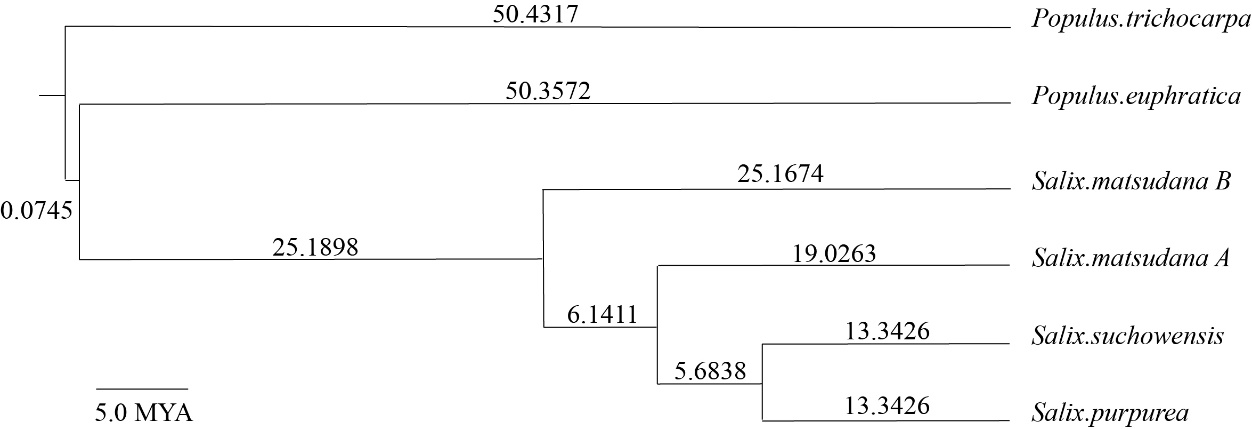
**

**Fig. S7 Phylogenetic relationships between the five tree species in Salicaceae**

**Table S1** Statistics of the second generation sequencing data in *S. matsudana*

| **Library** | **Data (**G**b)** | **Depth (×)** | **Q20 (%)** | **Q30 (%)** |
| --- | --- | --- | --- | --- |
| 270bp_1 | 35.97 | 57 | 96.63 | 93.18 |
| 270bp_2 | 42.53 | 68 | 96.88 | 93.11 |
| Total | 78.50 | 125 | --- | --- |

Note: Q20 and Q30 represent the proportion of bases with the quality value >20 and >30 in all the sequencing bases.

**Table S2** Statistics of the subreads length distribution from the third generation sequencing data of *S. matsudana*

| **Length (bp)** | **Num** | **Total length (bp)** | **Average length (bp)** |
| --- | --- | --- | --- |
| 0~2000 | 334,640 | 429,907,677 | 1,285 |
| 2000~4000 | 403,005 | 1,200,534,376 | 2,979 |
| 4000~6000 | 380,722 | 1,902,451,335 | 4,997 |
| 6000~8000 | 384,661 | 2,692,900,833 | 7,001 |
| 8000~10000 | 367,808 | 3,304,664,698 | 8,985 |
| 10000~12000 | 325,648 | 3,574,135,885 | 10,975 |
| 12000~14000 | 283,908 | 3,685,194,230 | 12,980 |
| 14000~16000 | 257,170 | 3,853,520,354 | 14,984 |
| 16000~18000 | 221,650 | 3,758,889,001 | 16,959 |
| 18000~ | 496,856 | 11,236,774,177 | 22,616 |
| Total | 3,456,068 | 35,638,972,566 | 10,312 |

**Table S3** Statistics of assembled genome sequences in *S. matsudana* (CEMGA Database)

| **Number of 458 CEGs* present in assemblies** | **% of 458 CEGs present in assemblies** | **Number of 248 highly conserved CEGs present** | **% of 248 highly conserved CEGs present** |
| --- | --- | --- | --- |
| 450 | 98.25% | 237 | 95.56% |

Note: Number of 458 CEGs* present in assemblies: the number of genes identified in the genome among the 458 CEGs in the CEGMA v2.5 database; % of 458 CEGs present in assemblies: the percentage of genes identified in the genome among the 458 CEGs in the CEGMA v2.5 database; Number of 248 highly conserved CEGs present: the number of genes identified in the genome among the 248 highly conserved CEGs in the CEGMA v2.5 database; % of 248 highly conserved CEGs present: the percentage of genes identified in the genome among the 248 highly conserved CEGs in the CEGMA v2.5 database.

**Table S4 Statistics of assembled genome sequences in *S. matsudana* (BUSCO Database)**

| **Complete BUSCOs** | **Complete single-copy BUSCOs** | **Complete duplicated BUSCOs** | **Fragmented BUSCOs** | **Missing BUSCOs** |
| --- | --- | --- | --- | --- |
| 1,313 | 314 | 999 | 46 | 81 |

Note: Complete BUSCOs: the number of complete genes in the BUSCO Database; Complete and single-copy BUSCOs: the number of complete single-copy genes in the BUSCO Database; Complete duplicated BUSCOs: the number of complete duplcated genes in the BUSCO Database; Fragmented BUSCOs: the number of fragmented genes in the BUSCO Database; Missing BUSCOs: the number of genes unpredicted in the BUSCO Database.

**Table S5 Statistics of the repeated sequences in *S. matsudana***

| **Type** | **Number** | **Length (bp)** | **Percentage (%)** |
| --- | --- | --- | --- |
| ClassI/DIRS | 23,299 | 12,858,816 | 1.97 |
| ClassI/LINE | 24,609 | 10,697,090 | 1.64 |
| ClassI/LTR | 16,462 | 5,372,871 | 0.82 |
| ClassI/LTR/Copia | 213,911 | 93,513,152 | 14.31 |
| ClassI/LTR/Gypsy | 227,569 | 100,397,354 | 15.36 |
| ClassI/PLE\|LARD | 226,830 | 68,012,354 | 10.41 |
| ClassI/SINE | 3,207 | 520,149 | 0.08 |
| ClassI/TRIM | 1,695 | 555,567 | 0.09 |
| ClassI/Unknown | 929 | 352,010 | 0.05 |
| ClassII/Crypton | 19 | 1,128 | 0 |
| ClassII/Helitron | 64,153 | 20,572,216 | 3.15 |
| ClassII/MITE | 741 | 180,006 | 0.03 |
| ClassII/Maverick | 1,273 | 237,328 | 0.04 |
| ClassII/TIR | 69,634 | 23,660,237 | 3.62 |
| ClassII/Unknown | 13,367 | 3,260,777 | 0.5 |
| PotentialHostGene | 40,566 | 11,796,414 | 1.81 |
| SSR | 6,185 | 3,303,809 | 0.51 |
| Unknown | 116,059 | 44,189,486 | 6.76 |
| Total | 1,050,508 | 300,418,768 | 45.97 |

Note: Type: the type of repeated sequences; Number: the number of repeated sequences; Length (bp): the total length of the predicted repeated sequences; Percentage (%): the proportion of repeated sequences to the whole genome sequences.

**Table S6 Statistics of functional annotation for the genes in *S. matsudana***

| **Database** | **Annotated number** | **100<=Protein length<300** | **Protein length>=300** | **Percentage (%)** |
| --- | --- | --- | --- | --- |
| COG Annotation | 22477 | 5804 | 16444 | 38.86 |
| GO Annotation | 38630 | 12106 | 25617 | 66.79 |
| KEGG Annotation | 20271 | 6431 | 13394 | 35.05 |
| TrEMBL Annotation | 57504 | 19448 | 35898 | 99.42 |
| nr Annotation | 57537 | 19480 | 35898 | 99.47 |
| All Annotated | 57546 | 19483 | 35899 | 99.49 |

Note: Percentage (%): the proportion of annotated genes to the total genes.

**Table S7 Statistics of gene families in the five tree species in Salicaceae**

| **Species** | **Total gene**  **number** | **Clustered gene**  **number** | **Total family**  **number** | **Unique gene**  **family number** |
| --- | --- | --- | --- | --- |
| *P. trichocarpa* | 41,335 | 33,300 | 19,190 | 495 |
| *S. purpurea* | 37,865 | 33,523 | 18,477 | 305 |
| *P. euphratica* | 30,612 | 28,971 | 17,066 | 86 |
| *S. suchowensis* | 26,599 | 23,820 | 15,853 | 51 |
| *S. matsudana* | 57,841 | 50,381 | 18,233 | 302 |

**Table S8** **GO enrichment analysis of the PAV differential genes in the A- and B-genome of *S. matsudana***

| **Ontology** | **GO term Level2** | **A-genome** | | **B-genome** | |
| --- | --- | --- | --- | --- | --- |
|  |  | **NO. of  genes** | **Precentage of genes (%)** | **NO. of  genes** | **Precentage of genes (%)** |
| Biological process | metabolic process | 3,693 | 54.28 | 4,231 | 55.66 |
|  | cellular process | 3,328 | 48.91 | 3,686 | 48.49 |
|  | response to stimulus | 1,601 | 23.53 | 1,684 | 22.15 |
| Cellular component | cell part | 3,012 | 44.27 | 3,403 | 44.76 |
|  | cell | 3,003 | 44.14 | 3,390 | 44.59 |
|  | organelle | 2,227 | 32.73 | 2,517 | 33.11 |
| Molecular function | catalytic activity | 3,590 | 52.76 | 4,085 | 53.74 |
|  | binding | 3,388 | 49.79 | 3,837 | 50.47 |
|  | transporter activity | 439 | 6.45 | 520 | 6.84 |
| Total gene |  | 6,804 |  | 7,602 |  |

**Table S9 Th**e **qRT-PCR primers used in this study**

| **Gene name** | **Primer sequence (5’-3’)** | **Primer sequence (5’-3’)** |
| --- | --- | --- |
| *Actin* | F:GTCAAGTTCTTTGCTTTCCTCC | R: CATCACAATCACTCTCCGACTA |
| *TCTP1* | F:GGGATGCTGTGGGAAGTTGA | R:ACAACCTTGACAGCCTGGTC |
| CYP71 | F:ATTGGGAGATGGCACTGGTG | R: GACACTTGGGCCAGCATTTG |
